# Suture Bridge Transosseous Quadriceps Tendon Repair for Spontaneous Quadriceps Tendon Rupture in Patients With End-Stage Renal Disease

**DOI:** 10.1016/j.eats.2022.08.022

**Published:** 2022-10-20

**Authors:** Surasak Srimongkolpitak, Bancha Chernchujit, Adinun Apivatgaroon, Pariwat Taweekitikul

**Affiliations:** a Orthopedic Department, Faculty of Medicine, Queen Savang Vadhana Memorial Hospital, Si Racha, Thailand; b Department of Orthopedics, Faculty of Medicine, Thammasat University, Bangkok, Thailand

## Abstract

End-stage renal disease with spontaneous quadriceps tendon rupture (QTR) is a specific condition that differs from classic QTR. The tissue quality of the quadriceps tendon (QT), the rupture site, the mechanism of injury, and the pathophysiology of the rupture mechanism all have an effect on conventional QT repair procedures, with a higher likelihood of rerupture or failed repair construction. We believe that our technique provides repair-site stability, strong repair construction, increased contact surface healing, and a reduced chance of rerupture after QT repair. Furthermore, in most patients who have end-stage renal disease with QTR, misdiagnosis and/or underestimation occurs, resulting in proximal retraction of the QT and poor results; however, this technique can be performed with alternative procedures such as augmentation or QT lengthening. The suture bridge transosseous QT repair technique relies on biomechanics knowledge for better stability. Suture bridge repair concept can achieve better healing of all layers of the QT until returning to normal activity with no disability and an improved quality of life.

Spontaneous quadriceps tendon rupture (QTR) is a common diagnosis in patients with end-stage renal disease (ESRD), rheumatoid arthritis, diabetes, systemic lupus, hyperparathyroidism, gout, and long-term steroid use. Spontaneous QTRs have been recorded in 3.2% of all QTR cases.^1^ We emphasize that secondary hyperparathyroidism, which is a factor that increases spontaneous QTR, will be a result of ESRD with hemodialysis.^1^, ^2^, ^3^ The occurrence of low-energy trauma with a simple fall (61.15%) or an indirect sudden and eccentric contraction of the extensor mechanism emphasizes that ESRD with subsequent hyperthyroidism is the key factor in weak and low-quality quadriceps tendons (QTs). After loss of the extension function of the affected knee, the patient immediately falls or is unable to stand on his or her own, according to the patient’s history.^4^

The clinical physical examination, on the other hand, is critical for diagnosis and treatment planning. QTR is a rare illness that can have a damaging effect and yield permanent disability if misdiagnosed. In cases of suspected QTR, a palpable suprapatellar gap is detected with lack of the extensor mechanism. Patella baja and calcific periosteal avulsion from the superior pole of the patella are observed on radiographs (Fig 1A and B). Magnetic resonance imaging is recommended to evaluate the degree of QT tearing, severity of proximal retraction, and associated soft-tissue injuries^5^^,^^6^ (Fig 1C and D). Because of several factors, including tendon quality, surgical repair techniques, underlying disease, and rehabilitation, we have found that QTR repair in ESRD patients yields poor outcomes and a higher risk of rerupture of the repair site.^7^

**Fig 1:**
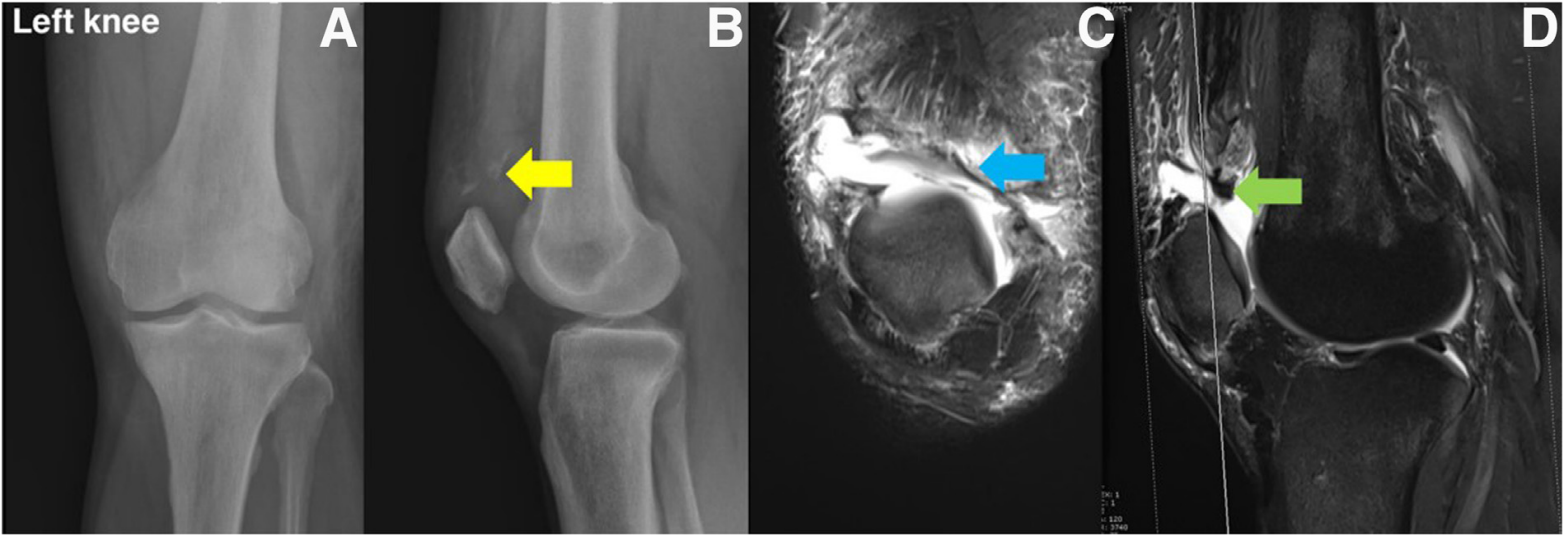
Radiographs of the left knee show downward displacement of the left patella with calcified deposits in the quadriceps tendon (yellow arrow): anteroposterior view (A) and lateral view (B). Magnetic resonance imaging of the left knee (T2 fat suppression (T2FS) in coronal plane) shows a rupture at the proximal patellar bone–tendon junction with extension to the medial and lateral retinaculum (blue arrow) (C) and shows complete disruption at the distal quadriceps bone-tendon junction and avulsion of the quadriceps tendon from the superior pole of the patella (green arrow) (D).

We suggest that our transosseous repair procedure has a low failure rate, particularly in terms of instrumentation pullout, as compared with suture anchor (SA) techniques. The suture bridge technique is used to attach the whole layer of the QT to the insertion of the upper pole of the patella. On the basis of the anatomy of the QT, strong suture repair technique, and strong suture material, our QT repair procedure is successful and yields good functional outcomes (Fig 2, Video 1).

**Fig 2:**
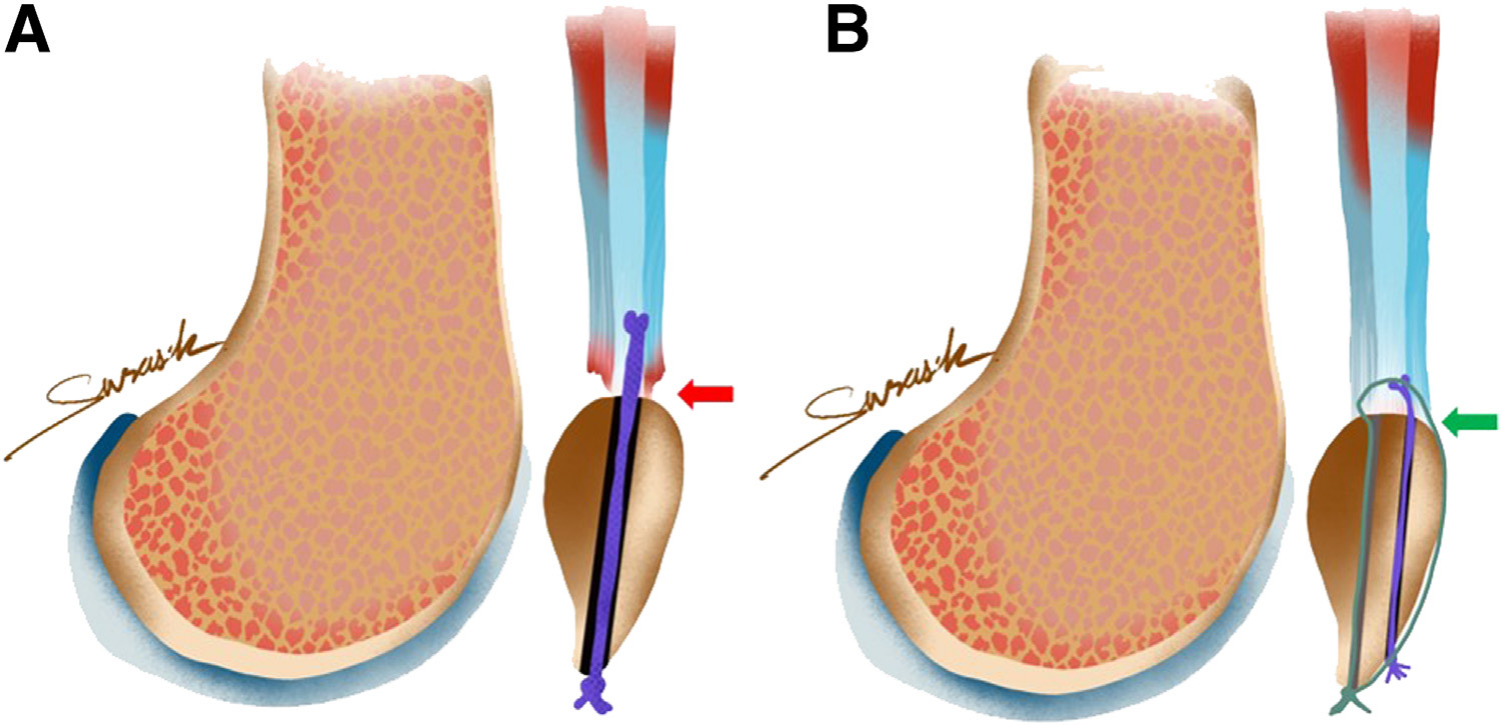
Comparative surface contact healing after quadriceps tendon repair. (A) With the conventional transosseous repair, some quadriceps tendon layer detachment (red arrow) is still observed. (B) With the suture bridge transosseous repair, all quadriceps tendon layers are completely attached at the superior pole of the patella (green arrow).

## Surgical Technique

### Patient Positioning

**Fig 3:**
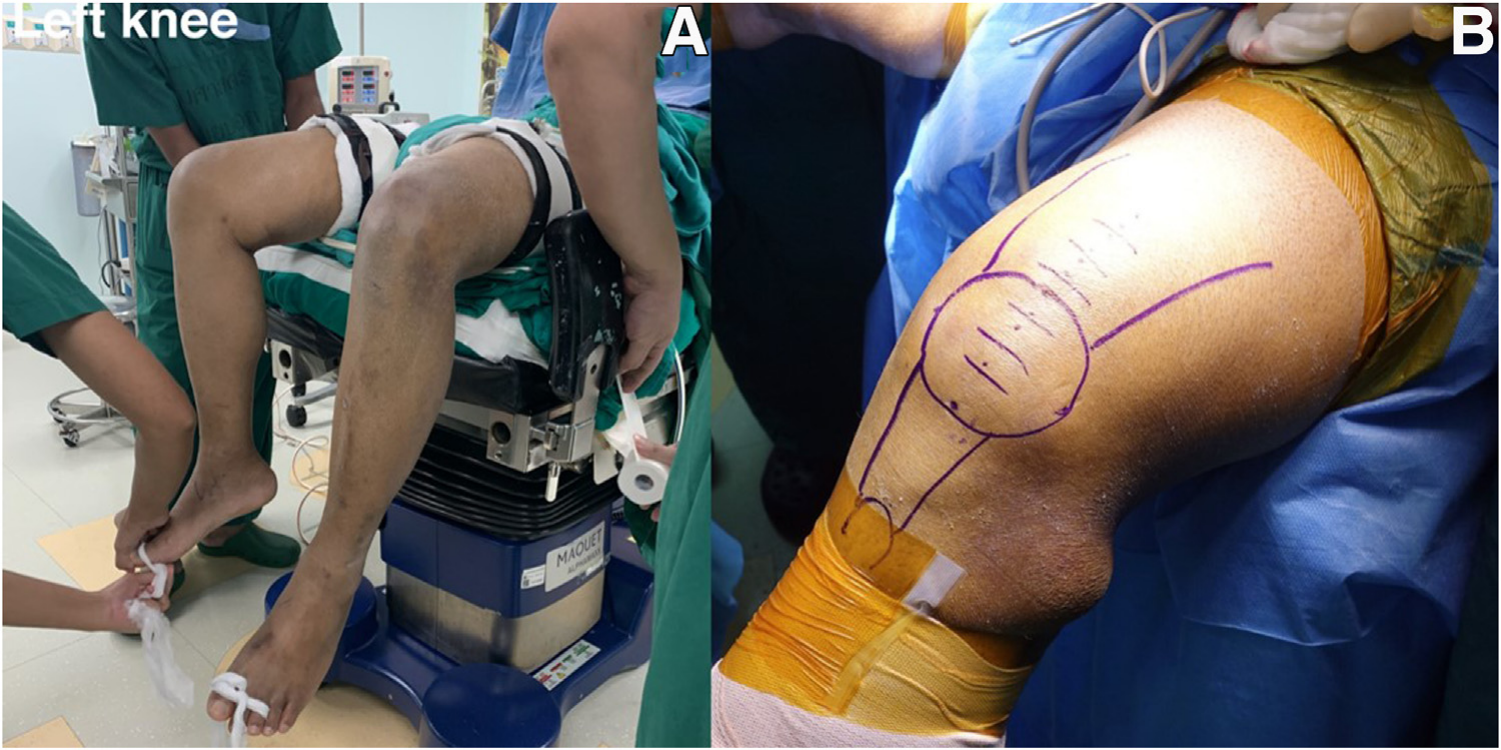
(A) The patient is in the supine position with the affected leg (left leg) hanging and the unaffected leg in the lithotomy position. (This patient had a diagnosis of bilateral quadriceps tendon rupture.) (B) Planning of the extensor mechanism anatomy is achieved with a midline incision.

The patient is positioned supine on the operating table, with the affected leg bent 90°, the posterior thigh placed in a long gel pillow holder, and the affected leg hanging over the side (Fig 3). The unaffected leg is abducted at the hip and placed in a lithotomy holder with sufficient padding. A well-padded pneumatic tourniquet is applied to the proximal thigh. The Esmarch bandage is exsanguinated before the tourniquet is inflated.

### Preparation of QT and Upper Pole of Patella

**Fig 4:**
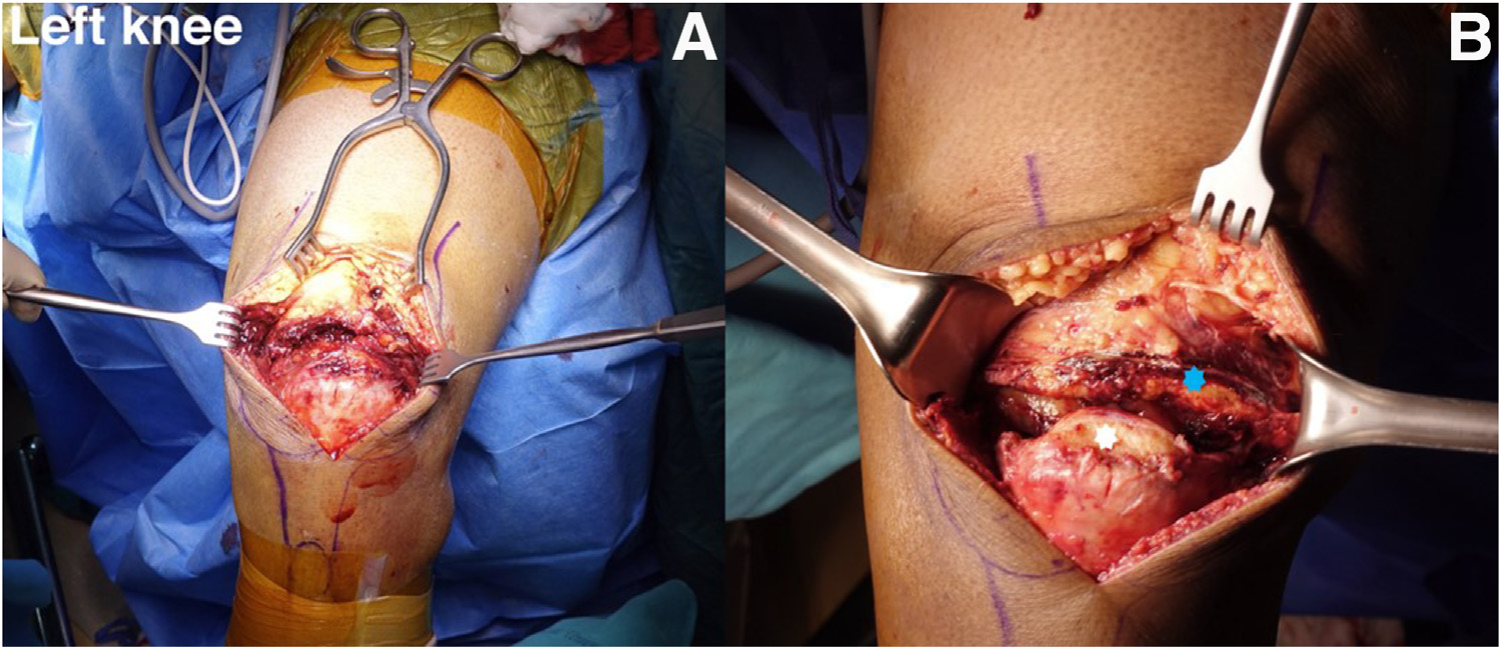
Intraoperative photographs of left knee. (A) A complete rupture between the quadriceps tendon and patella is exposed. (B) The color of the quadriceps tendon tissue stump indicates fibrous poor-quality tissue and the blood supply is poor (blue star); the superior pole of the patella is avulsed from the quadriceps tendon and is covered with the fibrous tissue (white star).

An 8-cm longitudinal incision is made from the proximal part of the QT tear site to the upper pole of the patella. This approach dissects the overlying QT tear and the proximal part of the patellar bone. The QT is exposed and removed the hematoma formation around the QT until the obvious anatomy of the QT is seen (Fig 4). Most patients will have extended tears of the medial and lateral retinaculum that must be exposed to prepare for the repair of both retinacular tears. The fibrous tissue and calcified periosteal tissue of the QT tear and upper pole of the patella should be debrided to enhance the tendon-to-bone healing process. The frayed soft tissue is debrided, the good-quality tendon ends are refreshed, and the upper pole of the patella is roughened. However, because patella alta will affect motion and patellofemoral contact pressure, the amount of debridement of the QT should not exceed 2 to 3 cm. Over-tensioning at the repair site will result in a reruptured QT repair; hence, over-resection of the QT must be avoided. The upper pole of the patella is roughened with a bone rongeur after part of the inactivated QT is removed. Microfracture is performed to improve growth factor regeneration.

### Placement of 5 Longitudinal Patellar Drill Holes

**Fig 5:**
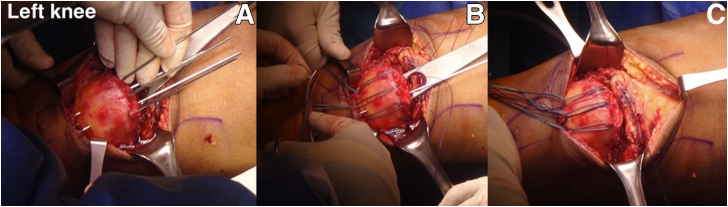
Intraoperative photographs of left knee. (A) The 5 longitudinal patellar drill holes are divided into 2 parts, consisting of 3 anterior patellar drill holes and 2 posterior patellar drill holes. (B, C) No. 2 Ethibond and polydioxanone are inserted into the holes to prepare for suture shuttling.

Five No. 2.0-mm pins are used to drill transosseous patellar tunnels longitudinally, consisting of 3 anterior and 2 posterior drill holes in the patellar bone (Fig 5). No. 2 Ethibond (Ethicon [Johnson & Johnson]) is inserted into the 3 anterior patellar drill holes to prepare for shuttling of FiberTape (Arthrex), which will be sutured to the QT. Polydioxanone (PDS; Johnson & Johnson) is inserted into the 2 posterior patellar drill holes to shuttle the No. 2 Ethibond.

### Suturing of Superficial and Middle Part of QT

**Fig 6:**
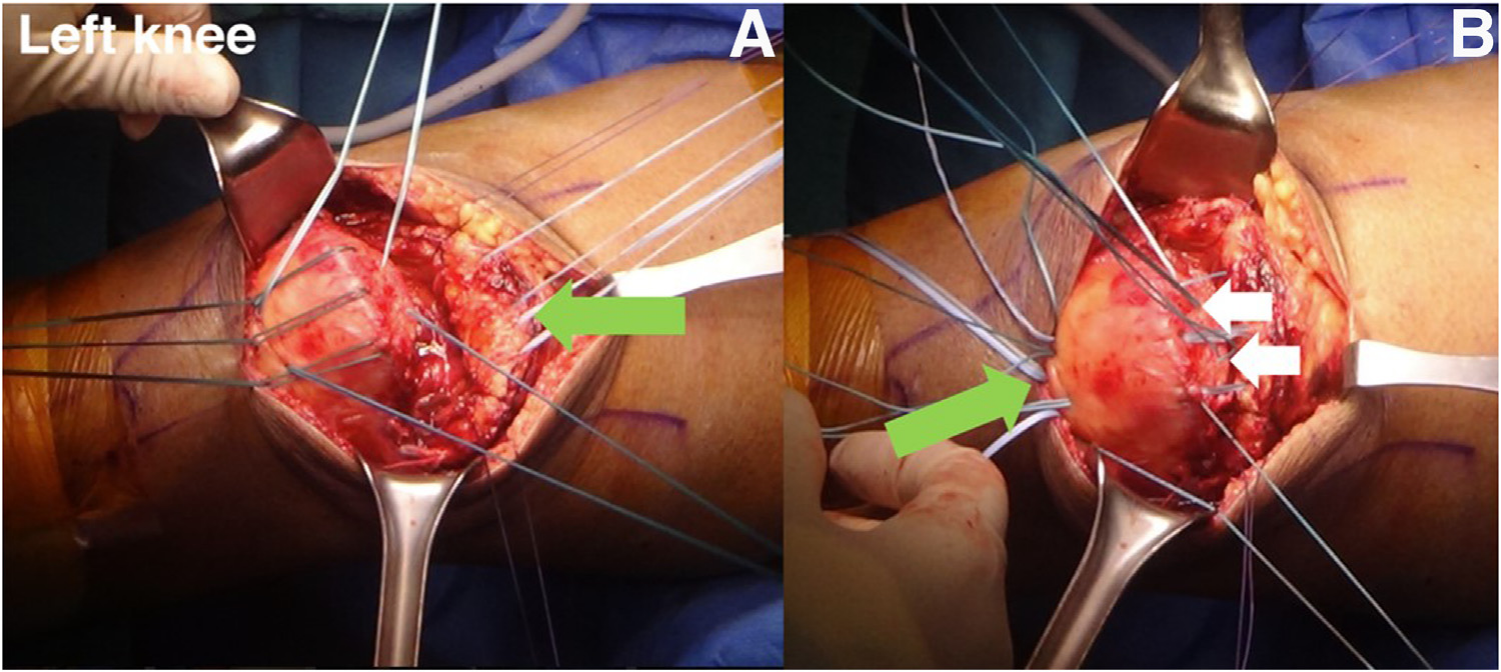
(A) The quadriceps tendon is sutured with 2 FiberTapes by 4 core strands (green arrow) with the Kessler repair technique. (B) The FiberTapes are shuttled into the anterior patellar drill holes (green arrow), and the 2 No. 2 Ethibonds are shuttled into each of the posterior patellar drill holes (white arrows).

Two FiberTape sutures are placed through the middle part of the torn tendon with the Kessler repair technique. The suture is placed at a distance of 2 to 3 cm proximal to the tear site. The 4 FiberTape suture limbs are shuttled through the 3 anterior drill holes of the patella where the No. 2 Ethibond is located. The No. 2 Ethibond is passed into the medial and lateral tunnel in the anterior of the patellar drill hole. The 4 suture limbs are passed through 3 parallel longitudinal tunnels within the patella, and the paired suture limbs are prepared to be secured on the inferior pole of the patella to secure the displaced middle part of the QT to its attachment site on the superior pole of the patella (Fig 6).

### Suturing of Deep to Superficial Part of QT

**Fig 7:**
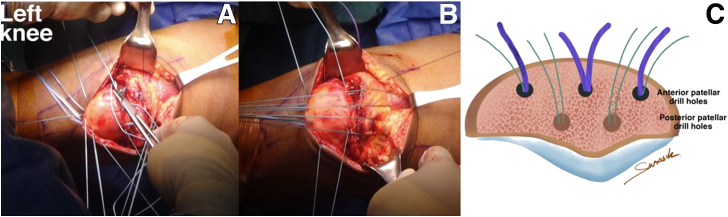
(A) All of the No. 2 Ethibonds from the posterior patellar drill holes are sutured in a retrograde manner from the deep to superficial quadriceps tendon. (B) The suture bridge from the posterior patellar drill hole is prepared to secure knot tying at the inferior pole of the patella. (C) Cross section of patellar bone, in which each anterior patellar drill hole comprises FiberTape (purple) and No. 2 Ethibond (green) and each posterior patellar drill hole comprises No. 2 Ethibond (green).

In the 2 posterior drilling tunnels of the patella, 2 No. 2 Ethibonds are shuttled with polydioxanone (PDS) and passed through each of the longitudinal transosseous drill holes in the patella. Four limbs of the No. 2 Ethibond suture are sutured in a retrograde manner from the deep part to the superficial part of the QT, where they are placed at a distance 2 cm proximal to the tear site. Sutures should be evenly spaced and should be evenly distributed along the total QT width (Fig 7).

### Suture Bridging of All Layers of QT

**Fig 8:**
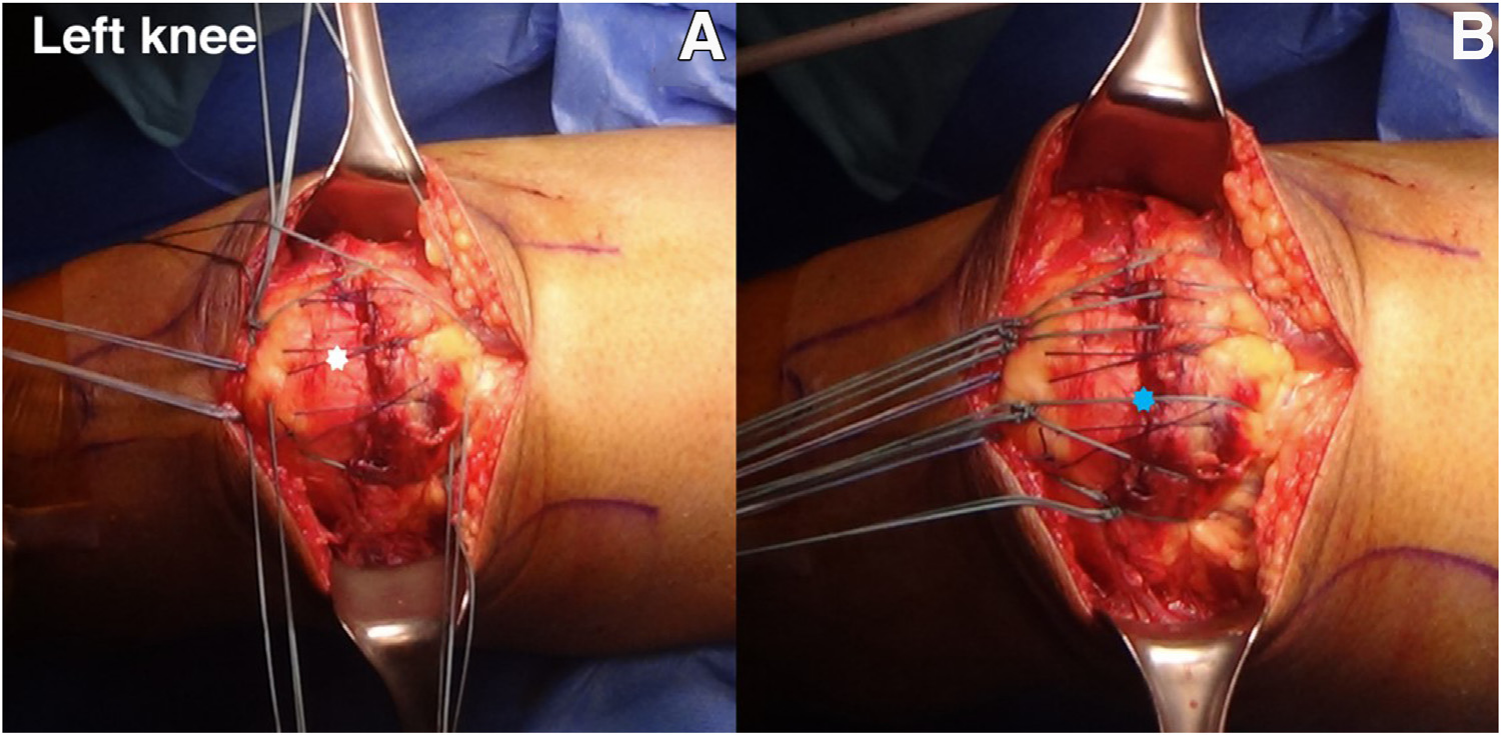
(A) The peritendinous quadriceps tendon is repaired with No. 1-0 Vicryl (white star) by a figure-of-8 suture technique. (B) All suture limbs (suture bridge technique) are tied at the inferior pole of the patella (blue star) with the left knee in full extension.

Sutured at the medial and lateral rim of the retinaculum via the medial and lateral anterior patellar drill holes by a figure-of-8 suture technique. The anterior peritendinous portion of the QT is sutured along the anterior of the patella with No. 1-0 Vicryl sutures (Ethicon [Johnson & Johnson]) (Fig 8A). The 4 FiberTape limbs are secured by a knot at the inferior pole of the patella with the knee in full extension. All the suture limbs from the posterior patellar drill hole are tied at the inferior patellar bone, which acts like a suture bridge. By means of this suture bridge construct, all layers of the QT will be reattached to the superior pole of the patella and rerupture of the QT will be prevented, especially in patients with ESRD who have poor tendon tissue quality (Fig 8B).

### Medial and Lateral Retinacular Repair

**Fig 9:**
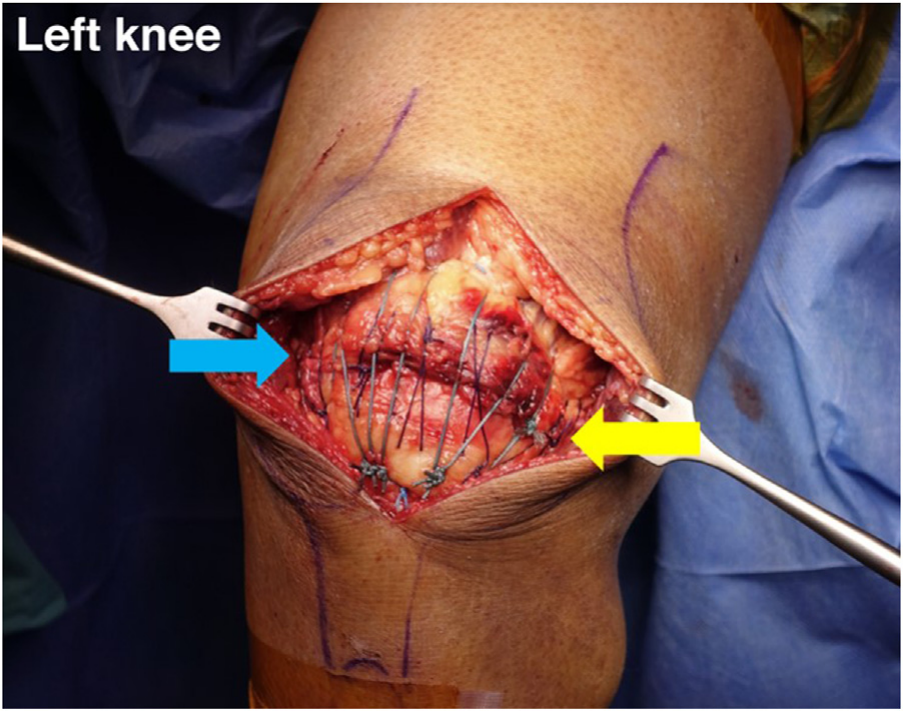
The stability of the suture construction is tested in 90° of knee flexion, and repair of both the medial retinaculum and lateral retinaculum of the left knee is performed with a figure-of-8 suture technique (blue and yellow arrows).

In most cases, the retinaculum is excessively torn both medially and laterally. The retinaculum should be repaired because it is one of the secondary extensor stabilizers of the knee. The retinaculum is sutured with No. 1-0 Vicryl by a figure-of-8 suture technique. The surgeon should confirm stability after the repair by bending the patient’s affected knee up to 90° intraoperatively without presenting tension to the sutured tendon and ensuring there is no gapping. The suture bridge repair technique be able to reduce tension across the entire layer of the QT (Fig 9).

### Postoperative Rehabilitation

A hinged knee brace is worn on both knees in full extension for a total of 8 weeks of immobilization. Wound care should be continually monitored with weekly follow-up visits for a duration of 3 weeks. During the immobilization period, the patient performs isometric quadriceps exercises. When the hinged brace is removed after 8 weeks, and then moves passively with progressive 20-degree knee flexion per week. The patient is instructed to undergo active motion rehabilitation of both knees for 10 to 12 weeks. At 3 months, the patient should be able to stand up and undergo proprioception training. Weight bearing is gradually increased until the patient is able to walk regularly. After the patient achieves a stable standing position and shows excellent proprioception, walking training can begin.

## Discussion

ESRD with long periods of hemodialysis and uremia, for example, is related to spontaneous tendon rupture, which affects collagen maturation and quadriceps muscle fiber atrophy. The pathophysiology of spontaneous QTR is produced as a result of systemic and local factors that affect blood flow and bone erosion at the patellar bone–tendon junction. Secondary hyperparathyroidism is a result of ESRD with hemodialysis, which might have developed into the spontaneous QTR.^8^^,^^9^

The QT is one of the thickest tendons, and its anatomy is complex and variable. The QT is divided into 3 layers based on its anatomy in the sagittal plane: the superficial layer, consisting of the rectus femoris; the middle layer, consisting of the vastus medialis and vastus lateralis; and the deepest layer, consisting of the vastus intermedius. The 3 layers’ tendinous fibers crisscross and connect along their borders to insert into the superior pole of the patella.^10^ According to Yepes et al.,^11^ QTRs are divided into 3 zones: zone 1 is between 0 and 1 cm, zone 2 is between 1 and 2 cm, and zone 3 is greater than 2 cm from the superior pole of the patella. Studies have shown that 35.6% of ruptures occur in zone 1, 41.4% occur in zone 2, and 12.1% occur in zone 3. Because hyperparathyroidism causes dystrophic calcifications and subperiosteal bone resorption and it can weaken the osteotendinous junction between the QT and the patella, zone 1 ruptures are most commonly found in patients older than 40 years and in patients with spontaneous ruptures with ESRD, which have a different pathophysiology than ruptures in zone 2, which has less vascular supply, in normal patients.^12^, ^13^, ^14^ The vascular density of the rectus femoris (superficial plane) is higher in the sagittal plane than the intermediate unit (the junction of the tendinous portions of the vastus medialis and vastus lateralis) and the deepest plane (vastus intermedius tendon). The deep region of the tendon (articular side) contributes to decreased vascularity in the deepest area of the tendon, making it at risk of rupture.^11^ The average thickness of the distal tendon is around 8 mm, with an average thickness of 16 to 18 mm at the patellar insertion site, according to anatomic dissection of the QT.^15^

The suture anchor (SA) technique and transosseous tunnel (TT) technique are the 2 major techniques for QT repair nowadays. The biomechanics and functional outcomes of the 2 techniques have been compared. There are no major functional differences between the TT and SA techniques. However, when compared with the TT technique, the SA technique has a higher rate of postoperative complications. Multiple reruptures occur in 3.7% of patients undergoing the SA technique. The failure rate in QTR was affected by rehabilitation compliance, health status and underlying medical comorbidities, age and activity level, as well as surgeon experience. Because patients with ESRD with hemodialysis have low bone quality and osteopenia, instrumentation pullout may occur more easily with the SA technique than with the TT technique. The use of patellar drill holes and the SA technique are 2 techniques for bone-tendon junction repair. The SA technique has the advantages of using a smaller incision, reducing the operational time, and reducing disturbances in the blood supply. The disadvantages of the SA technique in ESRD patients are the poor-quality bone base and that the pullout force is in the same longitudinal direction, which has a higher rate of fixation failure. Because of the higher complication rate, the SA’s reliability in terms of initial fixation strength has been a deterrent for many surgeons (implant pullout failure).^16^^,^^17^

When compared with constructs repaired by the TT technique, tendon displacement on initial cycling is consistently lower for SA fixation constructs, according to a systematic review.^18^ Between the SA and TT groups, no significant differences in construct stiffness or modes of failure were observed. Regarding modes of failure, knot slippage was the most common mode for both the TT and SA techniques. The modes of failure in the SA group were knot slippage (45.9%), suture tearing through tendon (23%), suture breakage (16.4%), anchor pullout from bone (11.5%), and other modes (3.2%). The modes of failure in the TT group were knot slippage (38.7%), suture tearing through tendon (32.3%), and suture breakage at knots (29.0%).^18^

It is suggested that patients undergo repair within 2 to 3 weeks in acute QTR. The surgical repair method can be accomplished side to side, and there is no proximal retraction of the QT, which may be performed during augmentation or lengthening and may lower patient satisfaction. If the acute QTR was repaired as fast as possible, the patient would achieve satisfactory results.^19^

Most chronic cases have poor quality tendons, which are affected by the combination of augmentation with repair of the QT procedure. The following augmentation techniques have been reported: biological tendon graft reinforcement and wire augmentation. The quadriceps muscle is likely to proximally retract until there is a gap in the QTR in delayed surgery cases. Therefore, quadriceps lengthening plays a role in combination with side-to-side repair.^20^

The principle of the reinforcement technique is to protect the suture from excessive tension in the case of more proximal retraction.^21^ A QTR requires a meticulous repair technique with possible biological augmentation, as well as a conservative rehabilitation protocol and intense physiotherapy.^22^ The postoperative care protocol after repair should include systematic specialist care, controlled secondary hyperparathyroidism, administration of vitamin D supplements, and consideration of parathyroidectomy with or without parathyroid gland autotransplantation. The systemic care will prevent the QT from rerupturing after repair.^23^

Nowadays, reruptures of the QT are still occurring in patients with ESRD with long-term hemodialysis. With the described repair technique, a device and stronger material have been invented and developed to improve the rate of healing (Video 1). This technique can achieve all the principles of tendon repair by improving contact surface healing of the entire layer of the QT to the upper pole of the patella; using transosseous patellar drill holes, which yields a lower rate of postoperative complications; using soft tissue -based preparation to promote the healing process of the bone-tendon junction; and using strong suture material that will provide better stability --thus decreasing the QT repair failure rate and resulting in a better QT repair (Tables 1 and 2).

**Table 1:** Pearls, Tips, and Pitfalls

Surgical Step	Pearls and Tips	Pitfalls
1. Preparation of QT and upper pole of patella	The healing process at the bone-tendon junction is enhanced. Poor-quality tendon tissue should be resected until healthy tendon tissue is visible. For better healing, the upper pole of the patellar bone is decorticated.	Because of patella alta and over-tensioning after repair, the QT should not be debrided and resected by >2-3 cm.
2. Placement of 5 longitudinal patellar drill holes	Each of the patellar drill holes must be spaced at least 1 cm from the other holes.	This technique may not be appropriate if the patellar thickness is <2 cm.
3. Suturing of superficial and middle part of QT	The middle to superficial part of the QT is sutured with FiberTape.	A more superficial suture would not be strong enough to prevent a higher chance of rerupture later.
4. Suturing of deep to superficial part of QT	All layers of the QT are reattached with increasing the contact surface healing. Sutures should be evenly spaced along the QT width.	The suture material does not penetrate into the joint when suturing the deep to superficial part of the QT in a retrograde manner.
5. Suture bridging of all layers of QT	The anterior peritendinous portion of the QT must be sutured to increase contact surface healing of the QT.	After the knot is secure, flexion of the knee must be used to confirm stability.
6. Medial and lateral retinacular repair	Once the knot has been tied securely, the stability of the knot should be tested by flexion of the knee. Side-to-side repair will improve the repair site’s stability and strength.	Over-tensioning of the retinacular repair should be avoided because it causes limited flexion of the knee.

QT, quadriceps tendon.

**Table 2:** Advantages, Limitations, and Disadvantages

Advantages
The suture bridge will increase contact surface healing of all the QT layers.
The suture material is strong enough to decrease the rate of rerupture of the QT.
Both primary and secondary extensor mechanisms are repaired.
Lower rates of construction failure and QT rerupture are achieved.
When compared with suture anchor techniques, our technique reduces postoperative complications.
The soft tissue– and bone–based preparation will promote QT healing, especially in patients with ESRD with hemodialysis.
In cases of excessive proximal retraction or chronic cases, this approach can use augmentation or lengthening.
Limitations and disadvantages
If the patellar thickness is <2 cm, this method should be avoided.
The use of several suture materials may increase the risk of infection.
Appropriate planning and suture management are essential.

ESRD, end-stage renal disease; QT, quadriceps tendon.

## Supplementary Data

ICMJE author disclosure forms

Video 1 The patient is positioned supine on the operating table, with the affected leg hanging in 90° of knee flexion. The midline incision is made around 8 cm from the proximal part of the quadriceps tendon (QT) to the patella. We debride the frayed soft tissue, refresh the good-quality tendon ends, and roughen the upper pole of the patella. Five No. 2.0 pins are used to drill transosseous patellar tunnels longitudinally, which comprise 3 anterior drill holes and 2 posterior drill holes in the patellar bone. No. 2 Ethibond is inserted in the 3 anterior patellar drill holes to prepare for shuttling of FiberTape, which will be sutured to the QT. Polydioxanone (PDS) is inserted in the 2 posterior patellar drill holes for shuttling of the No. 2 Ethibond. Two FiberTape sutures are placed through the middle part of the torn tendon by the Kessler repair technique. The distance of the suture is measured 2 to 3 cm proximal to the tear site. The 4 suture tails are passed through 3 parallel longitudinal tunnels within the patella. In the 2 posterior drilling tunnels of the patella, the 4 limbs of the No. 2 Ethibond suture are sutured in a retrograde manner from the deep part to the superficial part of the QT, where they are placed at a distance 2 cm proximal to the tear site. The anterior peritendinous portion of the QT is sutured with No. 1-0 Vicryl along the anterior of the patella with a figure-of-8 suture technique. The 4 FiberTape limbs are secured by a knot at the inferior pole of the patella with the knee in full extension. All the suture limbs from the posterior patellar drill holes are tied at the inferior patellar bone, which acts like a suture bridge. The retinaculum is sutured with No. 1-0 Vicryl by a figure-of-8 suture technique. After the repair, stability is confirmed when knee flexion of up to 90° can be achieved intraoperatively without causing tension.
